# Cryoglobulinemia due to Hepatitis C with Pulmonary Renal Syndrome

**DOI:** 10.1155/2013/278975

**Published:** 2013-06-05

**Authors:** Tariq Abdulkarim, Mohammad Saklayen, Jayson Yap

**Affiliations:** ^1^Wright State University, Dayton, OH 45409, USA; ^2^Dayton Veterans Affairs Medical Center, Dayton, OH 45428, USA

## Abstract

Cryoglobulinemia is an uncommon condition typically due to hepatitis C infection. Its clinical presentation is varied and often reflects deposition of immune complex and complement deposition. Renal compromise is observed in approximately one third of patients with mixed cryoglobulinemia and reports of concomitant pulmonary involvement are quite rare. We report a case of a patient who presented with pulmonary and renal manifestations of cryoglobulinemia with a serum rheumatoid factor over one hundred times the upper limit of normal and benefited from high-dose steroids and plasmapheresis in the acute setting.

## 1. Background

Cryoglobulinemia is a rare entity typically due to hepatitis C infection whose widely variable presentation can make prompt and accurate diagnosis difficult. Many of the features of cryoglobulinemia are manifestations of immune complex and complement deposition in the small- and medium-sized vessels [1–4]. The resultant vasculitis is observed in multiple organ systems with considerable variability between patients. The most common clinical features include purpura, weakness, and arthralgias; all of which are experienced by a vast majority of patients although perhaps only intermittently. Renal involvement is witnessed in 31% of patients with mixed cryoglobulinemia, and overall prognosis is worse for such patients [1]. Again, there is considerable variance in the severity of disease, from those who remain asymptomatic to those progressing to chronic renal insufficiency [4]. Pulmonary involvement in cryoglobulinemia is rare but had been documented as early as 1979 [5]. Typically, the patient has no signs or symptoms of lung involvement. Very rarely, the patient with cryoglobulinemia has concomitant alveolar hemorrhage. Ten case reports with such features have been published, and the presence of alveolitis seemed to portend a very poor prognosis. Of note, nine of those ten patients were also in renal failure at the time of alveolar hemorrhage. There was no specific therapeutic option that was shown to be of clear benefit. One patient did experience remission with plasmapheresis; however, five others did not [3].

## 2. Case Report

A forty-nine year old male presented to the emergency department complaining of hemoptysis of one-month duration. Past medical history included untreated hypertension and an episode of pericarditis two years earlier. The patient endorsed a 35-pack-year smoking history and consumption of 12 alcoholic beverages daily in addition to a remote history of intravenous drug use. Significant physical exam findings included a blood pressure of 168/95 mm Hg, bibasal crackles, epigastric tenderness to palpation, and 1+ bilateral lower extremity edema. No skin lesions or peripheral neuropathy were noted. Initial labs are shown in Table 1. Hepatitis C antibody was positive and antiglomerular basement membrane antibody was weakly positive as well at 1 : 20. ESR was found to be 70 mm/hr and ANCA was negative. Chest X-ray and renal ultrasound were unremarkable and a CT of the chest revealed diffuse interstitial changes.

In the context of markedly elevated rheumatoid factor, low C4, high C3, and high ESR in a patient with hepatitis C, cryoglobulinemia was suspected and subsequent serum cryoglobulin levels were positive. The patient was started on methylprednisolone 500 mg IV daily for suspected cryoglobulin-induced RPGN. Of note, the patient's hemoptysis resolved within a few days of admission. His kidney function worsened, with serum creatinine increasing steadily from 2.7 mg/dL [239 *μ*mol/L] on admission to a peak of 3.9 mg/dL [345 *μ*mol/L] on hospital day 14.

**Figure 1:**
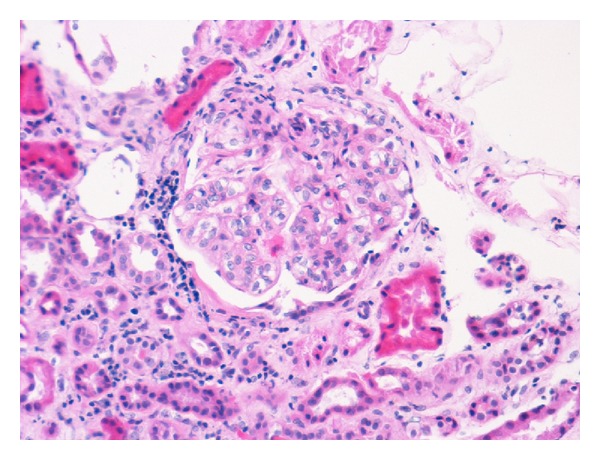
Kidney biopsy obtained on first admission showing hypercellular glomeruli with membranous proliferation.

He was started on daily plasmapheresis along with cyclophosphamide. Serum creatinine on the morning of the patient's first cycle was 3.0 mg/dL [265 *μ*mol/L]. In total, the patient underwent seven cycles of plasmapheresis 3 L every day. Serum creatinine improved to 1.5 mg/dL [133 *μ*mol/L] and a repeat rheumatoid factor was 717 IU/mL, a decrease of over 90% from the initial value. Renal biopsy was obtained and showed hypercellular glomeruli with membranous proliferation consistent with membranoproliferative glomerulonephritis type I (Figure 1). The patient was discharged the following day with plans to continue cyclophosphamide for the next four months along with a tapering dose of prednisone. Four months after discharge, rheumatoid factor was down to 233 IU/mL and both C3 and C4 levels were within normal limits. Cyclophosphamide and prednisone were discontinued and the patient was started on mycophenolate. This decision was made out of concern for the adverse effects of long-term use of the former. Approximately two weeks later, the patient presented to the ED complaining of hemoptysis of two-day duration in addition to decreased urine output. Blood pressure on admission was found to be 187/104 mm Hg. A CT Chest was obtained that showed consolidation and alveolar infiltrates in the left upper lobe. The patient was admitted and underwent three cycles of plasmapheresis with subsequent improvement in both his renal function and hemoptysis. On discharge, his serum creatinine had returned to 1.0 mg/dL [88 *μ*mol/L]. The patient was discharged on cyclophosphamide and prednisone and was later seen by gastroenterology and started on interferon and ribavirin therapy. 

The patient's course was complicated by leucopenia to the extent that his cyclophosphamide had to be discontinued. Approximately 12 months after his second admission, the patient was readmitted for suspected relapse. Labs were obtained and are noted in Table 1. He was given high-dose intravenous steroids with eventual normalization of his renal function. He was discharged on mycophenolate and prednisone at that time. He was unable to tolerate the mycophenolate due to leucopenia and the decision was made to discontinue it and start him on four weekly doses of rituximab in combination with low-dose prednisone. The patient did well on said therapy and was found to have a serum creatinine of 0.8 mg/dL [71 *μ*mol/L] and a urine protein/creatinine of 2.0 from a high of 8.6 prior to a few months.

## 3. Discussion

Cryoglobulinemia is a relatively rare disorder whose diagnosis can be challenging. This case illustrates an unusual presentation of the disease with concomitant renal and pulmonary involvement. In the limited number of previous case reports of patients with cryoglobulinemia and alveolar hemorrhage rheumatoid factor was reported as either positive or negative [3]. Serum rheumatoid factor on this patient came back at 7841 IU/mL, over one hundred times the upper limit of normal. Elevation of rheumatoid factor to this extent is extremely unusual and is worth reporting. Serum rheumatoid factor is typically increased in patients with HCV and mixed cryoglobulinemia [4]. Therapeutic options for the patient with cryoglobulinemia with renal and pulmonary involvement are limited. In addition to medical therapy, plasmapheresis was begun on this patient both at the initial presentation and the subsequent rehospitalization. Plasma exchange can significantly lower the amount of circulating cryoglobulins and is beneficial for patients with renal involvement [1]. Given the seemingly causal relationship between hepatitis C and mixed cryoglobulinemia, the treatment of the hepatitis is paramount [1]. Unfortunately, the long-term effect of such treatment on renal function in patients with cryoglobulinemic glomerulonephritis is currently unknown [6].

## Figures and Tables

**Table 1:** 

Lab	1st admission	2nd admission	3rd admission
BUN (mg/dL [mmoL/L])	166 [59.3]	112 [40.0]	50 [17.8]
Creatinine (mg/dL [*µ*moL/L])	2.7 [239]	2.9 [256]	1.7 [150]
Urine protein/Cr	1.76	2.57	8.6
Rheumatoid factor	7841 IU/mL	1900 IU/mL	68 IU/mL
C4 (mg/dL [g/L])	<2 [<0.02]	<2 [<0.02]	<2 [<0.02]
C3 (mg/dL [g/L])	88 [0.88]	68 [0.68]	113 [1.13]
